# Global numbers of infection and disease burden of soil transmitted helminth infections in 2010

**DOI:** 10.1186/1756-3305-7-37

**Published:** 2014-01-21

**Authors:** Rachel L Pullan, Jennifer L Smith, Rashmi Jasrasaria, Simon J Brooker

**Affiliations:** 1 Faculty of Infectious and Tropical Diseases, London School of Hygiene & Tropical Medicine, London, UK; 2 Institute for Health Metrics and Evaluation, University of Washington, Seattle, USA; 3 Stanford University School of Medicine, Stanford, CA, USA

**Keywords:** Soil-transmitted helminths, Ascaris lumbricoides, Trichuris trichiura, Hookworm, Disease burden, Disability-adjusted life years

## Abstract

**Background:**

Quantifying the burden of parasitic diseases in relation to other diseases and injuries requires reliable estimates of prevalence for each disease and an analytic framework within which to estimate attributable morbidity and mortality. Here we use data included in the Global Atlas of Helminth Infection to derive new global estimates of numbers infected with intestinal nematodes (soil-transmitted helminths, STH: *Ascaris lumbricoides, Trichuris trichiura* and the hookworms) and use disability-adjusted life years (DALYs) to estimate disease burden.

**Methods:**

Prevalence data for 6,091 locations in 118 countries were sourced and used to estimate age-stratified mean prevalence for sub-national administrative units via a combination of model-based geostatistics (for sub-Saharan Africa) and empirical approaches (for all other regions). Geographical variation in infection prevalence within these units was approximated using modelled logit-normal distributions, and numbers of individuals with infection intensities above given thresholds estimated for each species using negative binomial distributions and age-specific worm/egg burden thresholds. Finally, age-stratified prevalence estimates for each level of infection intensity were incorporated into the Global Burden of Disease Study 2010 analytic framework to estimate the global burden of morbidity and mortality associated with each STH infection.

**Results:**

Globally, an estimated 438.9 million people (95% Credible Interval (CI), 406.3 - 480.2 million) were infected with hookworm in 2010, 819.0 million (95% CI, 771.7 – 891.6 million) with *A. lumbricoides* and 464.6 million (95% CI, 429.6 – 508.0 million) with *T. trichiura.* Of the 4.98 million years lived with disability (YLDs) attributable to STH, 65% were attributable to hookworm, 22% to *A. lumbricoides* and the remaining 13% to *T. trichiura.* The vast majority of STH infections (67%) and YLDs (68%) occurred in Asia. When considering YLDs relative to total populations at risk however, the burden distribution varied more considerably within major global regions than between them.

**Conclusion:**

Improvements in the cartography of helminth infection, combined with mathematical modelling approaches, have resulted in the most comprehensive contemporary estimates for the public health burden of STH. These numbers form an important benchmark upon which to evaluate future scale-up of major control efforts.

## Background

Summary measures of population health are important to compare the relative importance of different diseases, to track differences in trends across countries and trends over time, and to provide a framework for evaluating the cost-effectiveness of interventions [1]. Estimating the burden of different diseases requires (i) reliable estimates of prevalence of each disease and (ii) an analytic framework within which to estimate morbidity and mortality attributable to each disease. In the case of intestinal nematodes (or soil-transmitted helminths, STH) several efforts have been made to provide worldwide prevalence estimates since those first assembled by Norman Stoll in 1947 [2-6]. The most recent, produced by de Silva *et al*. in 2003, suggested that despite marked declines in both the Americas and Asia for *Ascaris lumbricoides, Trichuris trichiura* and the hookworms (*Necator americanus* and *Ancylostoma duodenale*), little recent change had occurred in sub-Saharan Africa and STH still infected an estimated one billion people globally [7]. Inevitably, these numbers will have changed during the last decade: on the one hand, prevalence may have declined in some part due to improvements in living conditions and expansion of major deworming efforts; on the other hand, population growth may have increased the numbers infected.

A substantial hurdle when estimating numbers infected is the lack of quality data on infection prevalence [8]. In an effort to collate what data are available into a single resource, the Global Atlas of Helminth Infection (GAHI) was launched with the aim of describing the geographical distribution and prevalence of infection (http://www.thiswormyworld.org) [9,10]. As a first step, we previously used observed relationships between infection prevalence and environmental factors to delineate the global limits for each STH species, providing an essential basis for identifying the global population at risk of infection [11]. Building on this work, here we quantify the numbers infected within these limits for 1990 and 2010, and estimate potential global morbidity attributable to STH. The primary aim is to update global estimates of infection prevalence, thus providing reliable national and sub-national descriptions of variation in infection risk, highlighting major changes in the global picture of STH between 1990 and 2010, and identifying countries and regions where data are still notably lacking.

As a secondary aim, we use indirect methods to quantify populations at risk of morbidity attributable to infection. For STH, prevalence alone does not provide a useful measure of potential morbidity, as only a fraction of infections will be associated with ill health. Instead morbidity is related to the intensity of infection, with the most intense infections occurring in only a minority of infected individuals [12,13]. Given the lack of reliable methods for estimating disease directly, we build upon a mathematical modelling approach developed by Chan *et al.* and used in the original Global Burden of Disease (GBD) study [5] that exploits relationships between infection prevalence, mean intensity and potential morbidity [14,15]. We further develop these methods, which incorporate heterogeneity between communities and age classes to estimate potential morbidity at regional and global levels, by refining characterisation of geographical heterogeneity at smaller spatial scales. Numbers are generated fully within a Bayesian estimation framework, allowing propagation of uncertainty throughout the modelling process. The work informed estimates of disability-adjusted life years (DALYs) due to STH infection, as part of the GBD 2010 study [16].

## Methods

### Data assembly

We divide countries into 21 epidemiological regions, following the approach of the GBD study [1]. A total of 166 countries were classified as potentially endemic, including all countries in Asia (Central, east, south and south-east), Oceania, Latin America and the Caribbean, North Africa and the Middle East and sub-Saharan Africa. For each of these countries, digital boundaries obtained from 2009 version of the Administrative Level Boundaries project (SALB) [17] were overlaid on a population surface derived from the Gridded Population of the World version 3 [18] to estimate populations at the second administrative level (admin2, typically termed a district) for 2000. Age-specific population counts for 1990 and 2010 were subsequently generated by applying national, median variant, inter-censal growth rates and national demographic profiles [19]. Following procedures previously described, a series of biological limits were then applied to exclude populations living in areas without adequate survey data and where transmission is deemed biologically implausible based upon extreme aridity and thermal limits [11]. In total, 614 admin2 areas (2.4% of all admin2 areas considered, representing 125 million people) were classified as unsuitable for hookworm transmission, 713 (2.8%, 122 million people) for the transmission of *A. lumbricoides* and 899 (3.5%, 123 million people) for *T. trichiura*, and their population were subsequently excluded from all further analysis.

Data on the prevalence of helminth infections were abstracted from the ongoing GAHI project as described in detail previously [11,20]. When maintaining the GAHI database, periodic checks of complementary sources (including the Global Neglected Tropical Disease Database [21]) are carried out to ensure that available data data that complies with GAHI inclusion/exclusion criteria are included. For the current analysis, survey data were collated between 1980 and 2010, data older than this was used if no other data were available for a particular country. The abstracted dataset consisted of 6,651 quality-checked, geo-referenced estimates of infection prevalence. Where possible, surveys were located to a single latitude and longitude (i.e. point, 72.4% of data) [10]; where this was not possible surveys were geo-positioned to highest spatial resolution administrative area available, using the SALB boundaries database [17]. Table 1 summarizes the data by survey origin, spatial resolution, time period, age group and sample size. For the majority of countries with no data (20/38)^a^, transmission was excluded for both periods on a socioeconomic basis and a further three (Mauritius, Mayotte and Maldives) on the basis of comprehensive control. Seven countries in Oceania lacking data were assigned a regional mean prevalence based on 46 available surveys. ^b^The remaining eight countries with no data were assigned mean prevalence values based on observed data from neighbouring countries with similar eco-epidemiological situations (i.e. similar environmental and socio-economic conditions): areas suitable for transmission in Georgia, Iraq and Turkmenistan were assigned the same prevalence as neighbouring Iranian admin2, which were assumed to be eco-epidemiologically equivalent; Algeria data from Morocco; Timor Leste from Indonesia; PDR Korea from the Chinese Province of Jilin Sheng; and Tunisia and the Syrian Arab Republic from Turkey.

**Table 1:** Summary of available survey data as of end June 2011, by world region, spatial resolution, time period, age group and sample size

		**Spatial resolution**^**1**^				**Time period**			**Age group**					**Sample size**			
**World region**	**Total**	**Point**	**District**	**Province**	**National**	**Pre 2000**	**Post 2000**	**NR**^**2**^	**<5**	**SAC**	**Adults**	**Comm**	**NR**^**2**^	**<50**	**50-1000**	**>1000**	**NR**^**2**^
***Asia***	***1140***	-	863	248	29	437	883	240	3	529	34	256	318	265	595	120	160
Central Asia	**24**	-	7	11	6	2	23	1	0	11	0	10	3	0	17	7	0
East Asia	**69**	-	0	68	1	3	37	30	0	0	0	34	35	0	4	60	5
South Asia	**239**	-	195	41	3	99	171	62	2	150	6	66	15	59	160	9	11
Southeast Asia	**838**	-	661	158	19	333	682	147	1	368	28	176	265	206	414	74	144
***Latin America (LA) and the Caribbean***	***672***	-	523	69	80	262	264	265	6	157	3	296	210	46	329	148	149
Caribbean	**103**	-	27	23	53	37	82	11	1	35	0	34	33	3	66	34	0
Andean LA	**51**	-	38	7	6	32	43	8	0	37	0	10	4	12	33	3	3
Central LA	**213**	-	186	19	8	138	22	64	3	11	0	190	9	16	95	95	7
Southern LA	**21**	-	19	0	2	5	10	11	0	2	0	13	6	3	16	2	0
Tropical LA	**284**	-	253	20	11	50	107	171	2	72	3	49	158	12	119	14	139
North Africa and the Middle East	**163**	-	124	29	10	17	52	109	0	42	3	32	86	4	120	37	2
Oceania	**46**	-	39	0	7	12	39	0	0	35	0	11	0	9	30	7	0
***Sub-Saharan Africa (SSA)***	***4582***	4079	373	71	59	1989	3583	60	6	3700	19	854	4	1023	3521	39	0
Central SSA	**74**	18	48	7	1	49	69	0	0	68	2	4	0	2	70	2	0
East SSA	**2929**	2747	134	11	37	1204	2518	14	5	2417	9	494	4	610	2300	19	0
Southern SSA	**120**	45	75	14	3	76	71	15	0	98	0	22	0	51	68	1	0
West SSA	**1460**	1270	133	39	18	660	925	31	1	1117	8	334	0	360	1083	17	0
***TOTAL***	***6604***	4080	1922	417	185	2717	4821	674	15	4463	59	1449	618	1347	4595	351	311

^1^Resolution of geo-location: Point, latitude and longitude. Data for SSA that could not be located to a single point was still used to inform within-administrative area prevalence distributions.

^2^NR = not reported. For unreported year, data was used in both 1990 and 2010 estimates, for age group data was assumed to reflect community prevalence, for sample size individuals tested were assumed to be 100. SAC; School-aged children. Comm; community – includes all ages or crosses age group categories.

### Age-stratified mean prevalence estimation at sub-national levels

The approaches used to map mean prevalence of infection within the boundaries of transmission differed by region, determined by the progress in control, environmental associations and data availability considerations. For countries within sub-Saharan Africa – where detailed data were lacking for several countries but where relationships between infection patterns and environmental factors were clearer – a model-based geostatistical (MBG) space-time modelling framework was used to predict the prevalence of each infection across the continent, following the approach of Hay *et al. *[22]. For all other world regions, empirical estimates were generated directly from the data.

**Figure 1:**
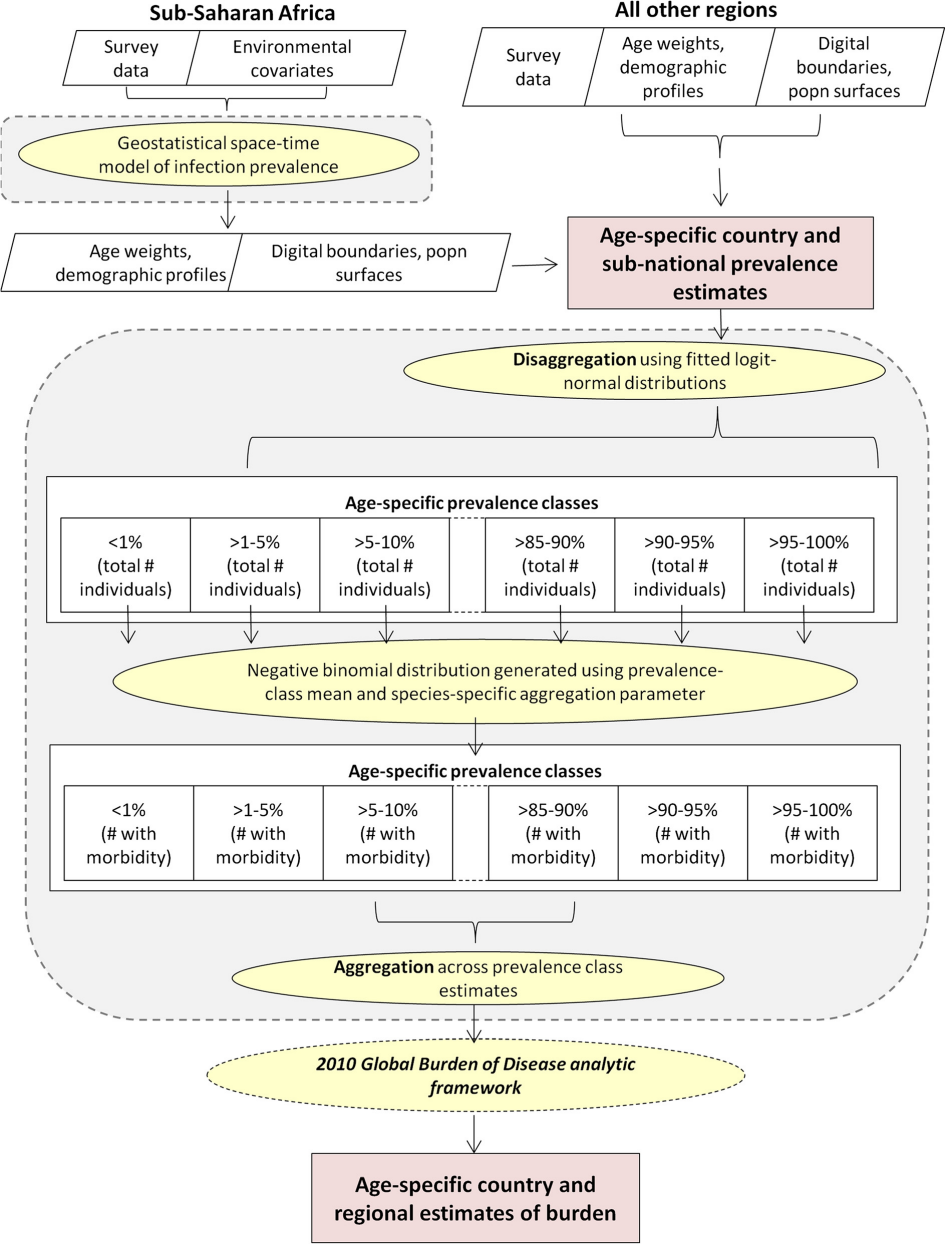
**Schematic of methods used to estimate populations at risk of morbidity.** Age-specific prevalence estimates were generated using geostatistical modelling (SSA) or on an empirical basis (all other regions). Geographical variation was approximated using modelled logit-normal distributions, and numbers exceeding burden thresholds estimated using negative binomial distributions. These results were fed into the 2010 GBD framework to estimate years lived with disability (YLD) and Disease Adjusted Life Years (DALYs). Steps contained within shaded grey areas are completed within a Bayesian framework.

The approach adopted for sub-Saharan Africa is predicated on the role of environmental factors in influencing the large-scale geographic distributions of infection, in the absence of substantive control measures [23-26]. Full details on the methodology are provided in Additional file 1. In brief, within the Bayesian MBG framework the probability of being infected at each survey location was modelled as a function of nearby survey data (weighted according to spatial and temporal proximity) and socio-environmental covariates (land surface temperature and normalized differenced vegetation index [27], population density [28] and survey-type (school-based vs. community-based)). This was followed by a prediction stage in which samples were generated from the posterior distribution of infection prevalence in children aged 5 to 14 years in 2010 at each prediction location on a 5 × 5 km grid. Both the inference and prediction stages were coded using Python (PyMC version 2.0) using a bespoke Markov chain Monte Carlo (MCMC) algorithm [29]. Subsequently, at each prediction location prevalence in children aged 0 to 5 years and adolescents and adults aged ≥15 years were estimated based on age-prevalence weights initially proposed by Chan *et al. *[30] and shown in Table 2. The predictive surface was overlaid with administrative boundary and population data described above to determine overall and age-specific mean prevalence rates for each admin2 area. These admin2 mean prevalence estimates were then handled using the same methodology as that used for all other world regions, as shown in Figure 1. As a single point prediction process was used, aggregated estimates of uncertainty were not valid. Therefore, only the estimated district mean prevalence estimates were assigned to each district, and no estimation of uncertainty.

**Table 2:** Parameters used for modelling the age distribution of infection, and the distribution of high intensity infections

**Species**	**Age class (in years)**	**Age weight for prevalence**	**Aggregation parameter ( *k *)**	**Morbidity threshold**^**1**^		
				**Light intensity**^**2**^	**Medium intensity**	**High intensity**
Hookworms	0-5	0.75	*f*(prevalence)^3^	1	2000	4000
	5-10	1.2	*f*(prevalence)^3^	1	2000	4000
	10-15	1.2	*f*(prevalence)^3^	1	2000	4000
	15 plus	1.0	*f*(prevalence)^3^	1	2000	4000
*A. lumbricoides*	0-5	0.75	0.54	-	90	250
	5-10	1.2	0.54	-	130	375
	10-15	1.2	0.54	-	180	500
	15 plus	1.0	0.54	-	180	500
*T. trichiura*	0-5	0.5	0.23	-	50	105
	5-10	0.75	0.23	-	75	160
	10-15	0.9	0.23	-	100	210
	15 plus	1.0	0.23	-	100	210

^1^Intensity of infection for hookworm is expressed in terms of eggs per gram of faeces, for *A. lumricoides* and *T. trichiura* in worm burden. ^2^There is insufficient evidence to quantify the impacts of light intensity infection for *A. lumbricoides* and *T. trichiura*, and as such no disability weighting is applied to this group. ^3^Aggregation parameter is estimated as a function of prevalence (p) : k = 0.12p + 0.175p^2^ + 0.0008.

In contrast to sub-Saharan Africa, an increased proportion of available data for all other world regions originates from nationally representative surveys (e.g. [31-34]). In regions outside sub-Saharan Africa, environmental relationships are also likely to be more ambiguous, especially in the sub-tropics [11], due to both the increased seasonality and the modifying influence of improvements in socioeconomic conditions and sustained, large-scale control. In addition, most data in these regions could only be assigned to an administrative area, rather than a point, limiting the potential usefulness of predictive MBG modelling approaches. For this reason, empirical estimates were generated directly. Mean prevalence estimates were initially aggregated at an admin2 level (representing on average 500 km^2^ and 30,500 people), as this was considered of sufficient geographical resolution to capture large-scale variation in the distribution of both worms and humans. First administrative (admin1, typically a province or region) or national estimates were applied to admin2 without data for those countries without geographically comprehensive survey data. Estimates were generated for four age groups, weighted according to well-established age patterns shown in Table 2. For those countries without geographically or temporally comprehensive survey data specific decisions were made on a country-by-country basis as outlined above and are detailed in Additional file 2.

Mean prevalence estimates were generated for the two time periods of the GBD 2010 study: 1990 and 2010. For countries in sub-Saharan Africa, the 2010 predictions were applied to both time periods, based on the assumption of no sustained, geographically extensive control programmes, and an observation of no consistent temporal trend for the region. For other world regions, 1990 estimates are based on survey data from 1980–1999, whilst 2010 estimates are based on data from 2000–2010. Finally, prevalence estimates were adjusted for a limited number of countries that have recently implemented large-scale treatment campaigns, through either school-based deworming programmes or community-based lymphatic filariasis elimination programmes. Information about the coverage of these campaigns was assembled from relevant sources [35-40] and adjustments were made that reflected treatment coverage levels over the past five years using a mathematical model of transmission dynamics implemented via the computer programme EpiWorm [41,42]. This program allows the user to specify the local epidemiological data and the coverage of school- and community-based chemotherapy over a series of year, and calculates predicted reductions in prevalence based on these data. For implicated countries outside sub-Saharan Africa, 2010 estimates were reduced to reflect the control measures; for countries within sub-Saharan Africa, where 2010 prevalence estimates included a temporal component and better reflected post-control prevalence, 1990 estimates were adjusted accordingly. Further details are provided in Supplementary Materials 2. Finally, for presentation purposes prevalence of any STH was estimated using a simple probability model, incorporating a small correction factor to allow for non-independence between species, following the approach of de Silva and Hall [43].

### Estimating populations at risk of morbidity

The risk of potential morbidity is based on the empirical observation that there is some worm burden threshold above which morbidity is likely to occur [15]. In the previous round of the GBD study, age-specific morbidity thresholds were defined that assumed risk of morbidity occurred at higher worm counts with increasing age [5,14]. The frequency distributions of worm counts, and thus the numbers exceeding these thresholds, were estimated using negative binomial distributions that assumed general species-specific aggregation parameters. In our analysis, hookworm burden was related to intensity of infection as expressed by quantitative egg counts using defined thresholds (light = 1–1,999 epg; medium = 2,000-3,999 epg; heavy = over 4,000 epg) and applied across all age-groups. This is because (i) most literature on the health impact of STHs expresses results in these terms [44,45], and (ii) empirical data on egg counts were available to better quantify the aggregation parameter. Exploratory analysis of intensity data from Brazil [46], Kenya [47] and Uganda [48] suggested that *k* varies as a quadratic function of prevalence; consequently a fitted value for *k* was used as shown in Table 2. In contrast, we did not have sufficient contemporary, high prevalence *A. lumbricoides* and *T. trichiura* egg count data to redefine relationships for these two infections across all settings, and so the original thresholds and aggregation parameters were used for the current analysis (Table 2).

The non-linear relationship between prevalence and infection intensity dictates that the proportion of the population at risk of morbidity will be disproportionately greater in communities where the prevalence of infection is highest. The framework employed to estimate numbers at risk of morbidity thus needed to account for geographical (within admin2) heterogeneity and over-dispersion of infection intensity [5,14]. In brief, for each survey *k* in admin2 *j* in country *i* within and between admin2 variance was modelled within a Bayesian framework using a simple nested linear mixed model:

$$\begin{array}{l} \mathrm{logit}\left({\hat{p}}_{\mathrm{ijk}}\right)=\mathrm{logit}\left(p_{i}\right)+\epsilon_{\mathrm{ijk}}+\theta_{\mathrm{ij}},\mathrm{logit}\left(p_{i}\right)=\mu_{1}\\ \;+u_{0i},u_{0i}\sim N\left(0,\sigma_{b}^{2}\right),\epsilon_{\mathrm{ijk}}\sim N\left(0,\sigma_{w1}^{2}\right),\\ \;\theta_{\mathrm{ij}}\sim N\left(0,\sigma_{w2}^{2}\right) \end{array}$$

where the parameter $\sigma_{w1}^{2}$ represents within admin2 variation in infection prevalence, $\sigma_{w2}^{2}$ represents within country variation and $\sigma_{b}^{2}$ between country variation. The variance parameters $\sigma_{w1}^{2},\sigma_{w2}^{2}$ and $\sigma_{b}^{2}$ were assigned semi-informative gamma priors [49] and μ1 a non-informative normal prior (mean 0 and precision 1x10-6). This specification was chosen because examination of within-admin2 heterogeneity for admin2 areas with >10 available unique surveys points suggested that, although distributions differed between worm species, all three species were highly skewed and best described by logit-normal distributions.

After an initial burn in of 10,000 iterations, the model was run for a further 10,000 iterations with thinning every ten. At each stored iteration, the age-specific distribution of prevalence amongst populations in each admin2 region was estimated based on logit(pi) and $\sigma_{w1}^{2}$. A negative binomial distribution was then applied to each 5 percentile using species- and age-specific aggregation parameters (k), and the number of individuals with more than the threshold worm/egg count calculated (see Table 2). The estimated numbers of individuals above threshold counts were then summed over all 5 percentiles to estimate age-specific populations at risk of morbidity at admin2, national and regional levels. Uncertainty in the degree of within admin2 heterogeneity, and its impact upon estimated populations at risk of morbidity, was thus propagated throughout the modelling process.

### Estimation of disease burden

As a summary measure of disease burden we use a DALY framework, which incorporates both year of life lost from premature death (YLL) and years of life lived with disability (YLD) into a composite estimate. This work was conducted by the core modelling team of the GBD 2010 study [50,51] and full details of the methodology are provided in [16]*.* In brief, disability weights from the GBD Disability Weights Study [52] were applied to each category of infection intensity to estimate YLDs as outlined in Table 3. These disability weights are assigned to four major sequelae attributed to STH infection: abdominopelvic problems, symptomatic infection, wasting and anaemia – the latter applying to hookworm only.

**Table 3:** Description of disability weights for each soil-transmitted helminth species

**Species**	**Sequalae and disabling consequences**	**Infection intensity**	**Disability weighting**
*A. lumbricoides*	Symptomatic infection	Heavy	0.0296
	Wasting	Heavy	0.1245
	Mild abdominopelvic problems	Medium	0.0108
*T. trichiura*	Symptomatic infection	Heavy	0.0296
	Wasting	Heavy	0.1245
	Mild abdominopelvic problems	Medium	0.0108
Hookworm	Mild anaemia	All	0.0041
	Moderate anaemia	All	0.0056
	Severe anaemia	All	0.1615
	Wasting	Heavy	0.1245
	Mild abdominopelvic problems	Medium	0.0108

Abdominopelvic problems and symptomatic infection are considered as contemporaneous disabling consequences that are assumed to occur in 100% of individuals who harbour worm burdens above the higher threshold, and persist for the duration of infection [5,53]. To estimate the wasting attributable to heavy infection, a two stage approach was adopted. First, the prevalence of wasting among children under five years old was independently estimated using available data. Second, the prevalence of wasting due to STH was calculated by shifting the 2006 WHO reference population weight-for-height distribution according to the product of (i) the proportion of individuals harbouring worm burdens over the higher threshold and (ii) the average shift in weight-for-height per case of heavy STH infection, based on a meta-analysis of randomized controlled trials of mass deworming [54]. The pooled effect across identified studies was a change in weight-for-height z-score per affected individual of 0.4938. Finally, the overall prevalence of wasting attributable to heavy STH infections due to each species was calculated based on their relative distribution.

Anaemia outcomes attributable to hookworm infection were estimated using a similar approach: mean haemoglobin shift caused by hookworm were taken as the pooled results of treatment trials, estimated as 2.08 g/l [55], and the fraction of anaemia burden attributable to hookworm calculated based on the prevalence of anaemia in the general population, which was again independently assessed [56].

Deaths from STH are all attributable to heavy *A. lumbricoides* infection, and are primarily due to intestinal obstruction and biliary or pancreatic disease in children under 10 years of age [57]. YLL for *A. lumbricoides* were modelled using a negative binomial regression incorporating ln-transformed age-standardised *A. lumbricoides* prevalence, age and sex as primary covariates and using vital registration, verbal autopsy and surveillance data from the GBD cause of death database as the outcome. This structure is well-suited to model rare outcomes with sparse data. YLL estimates were generated for each five-year age-group by country, sex and time period, before aggregating using national demographic profiles [19]. Finally, *A. lumbricoides* mortality (and all other causes of mortality generated as part of the GBD 2010 study) were corrected to sum to the estimated all-cause mortality rate.

## Results

### Data availability

Data included in this analysis are summarised in Table 1. In total, we identified 4,079 point prevalence estimates from 2,803 spatially unique locations for inclusion in the MBG predictive model for sub-Saharan Africa. Data coverage was highly clustered: 50% of available data originated from just three countries (Kenya, Uganda and Cameroon), eight countries had fewer than 10 data-points (Central African Republic, Congo, Mauritania, Mozambique, Senegal, Sierra Leone, Somalia and Togo), and for a further 10 countries no data were available (Angola, Botswana, Cape Verde, Comoros, Equatorial Guinea, Gabon, Guinea-Bissau, Lesotho, Liberia and Swaziland). Overall, 58% of surveys were conducted since 2000 and 87% surveyed school-aged or pre-school aged children.

For other world regions, data were available for 2,012 locations from 82 of the 120 included countries: 1,519 data-points could be geolocated to the admin2 level, 355 to the admin1 level and 138 at the country level only. The best represented region outside sub-Saharan Africa was Asia (excluding Central Asia), for which we were able to assemble data from both time periods (pre and post 2000) for 16 of 34 countries. Large representative national or sub-national surveys were available for a number of Asian countries including the People’s Republic of China, the Republic of Korea and Indonesia although notably for India data were lacking, with only 129 identified surveys, the majority of which (80%) were point prevalence estimates. Data coverage for Latin America were geographically clustered, with substantial data originating from well-characterised at-risk regions in Brazil (281 surveys), Honduras (28 surveys) and Venezuela (146 surveys) and few data points for the rest of Central, southern and Andean Latin America. The relatively sparse data for North Africa and the Middle East and Central Asia, as well as the island nations of Oceania and the Caribbean, primarily originated from point prevalence estimates. Although Yemen was an exception with 62 available surveys, in general countries in these regions rarely had data from more than 10 surveys; over 80% (67 of 79) had fewer than five data points and no data were available for 30 countries. Overall, 35% of the available data for Latin America and the Caribbean was collected between 2000 and 2010, comparing with 51% from Asia, 69% from North Africa and the Middle East and 74% from Oceania.

### Mean prevalence estimates

**Figure 2:**
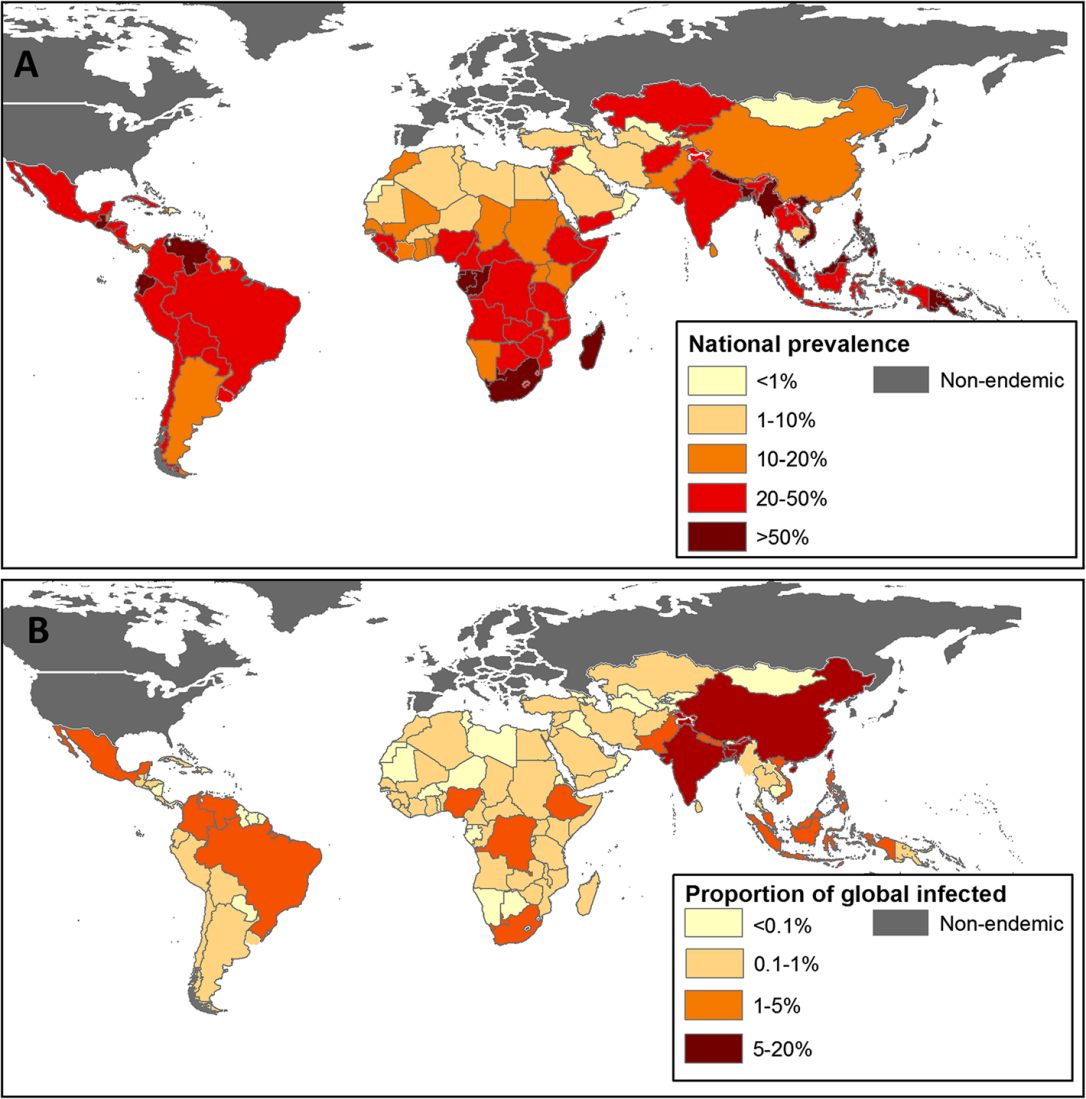
**Distribution of any STH infection in 2010. (A)** The combined prevalence of any infection, based on geostatistical models for sub-Saharan Africa and available empirical information for all other regions. **(B)** The proportion of the global population infected (1.45 billion) by country.

Regional numbers infected and prevalence estimates for 2010 are provided in Table 4. Globally, our estimates suggest that 438.9 million people (95% Credible Interval (CI), 406.3 – 480.2 million) were infected with hookworm in 2010, 819.0 million (95% CI, 771.7 – 891.6 million) with *A. lumbricoides* and 464.6 million (95% CI, 429.6 – 508.0 million) with *T. trichiura.* Almost 70% of these infections occur in Asia. Figure 2 emphasises this point, highlighting the high proportion of total individuals infected with one or more STH residing in the People’s Republic of China (18%) and India (21%). By contrast, the three most populous nations in sub-Saharan Africa (Nigeria, Ethiopia, Democratic Republic of the Congo) in total account for only 8% of global STH infections. In 58 countries, hookworm prevalence exceeds 20% for at least one sub-national area (admin2 or admin1), compared with 47 for *A. lumbricoides* and 45 for *T. trichiura.* In total, we estimate that 1.01 billion school-aged children live in admin2 areas where prevalence of any STH is expected to exceed 20% (16% of these in sub-Saharan Africa, 71% in Asia, 13% in Latin America and the Caribbean).

**Table 4:** Estimates of global numbers infected with soil-transmitted helminths in 2010, by region

**REGION**	**Total population (millions)**	**Infected Populations in millions (95% CI**^**1**^**)**			**Overall prevalence (95% CI)**		
		**Hookworm**	***A. lumbricoides***	***T. trichiura***	**Hookworm**	***A. lumbricoides***	***T. trichiura***
**Asia**	**3736.7**	**281.8 (249.5-318.5)**	**589.0 (524.4-660.3)**	**282.3 (248.5-323.5)**	**7.5% (6.7-8.7%)**	**15.8% (14.5-17.7%)**	**7.6% (6.6-8.7%)**
Central Asia	80.7	0.1 (0.01-0.2)	6.0 (5.1-6.9)	0.1 (0.2-.25)	0.1% (0.0-0.3%)	7.4% (6.4-8.5%)	0.1% (0.0-0.3%)
East Asia	1424.4	64.5 (44.9-87.3)	158.4 (124.7-194.1)	66.2 (41.9-93.5)	4.5% (3.3-5.5%)	11.1% (8.8-13.6%)	4.6% (3.0-6.6%)
South Asia	1621.1	140.2 (117.2-173.0)	297.8 (263.8-345.4)	100.7 (80.3-129.8)	8.7% (5.2-6.6%)	18.4% (16.3-21.9%)	6.2% (5.0-8.0%)
Southeast Asia	610.5	77.0 (69.2-84.9)	126.7 (116.0-137.4)	115.3 (106.8-125.3)	12.6% (11.3-13.9%)	20.8% (19.0-22.5%)	18.9% (17.5-20.5%)
**LAC**	**586.0**	**30.3 (25.5-35.5)**	**86.0 (78.2-95.6)**	**72.2 (66.0-80.0)**	**5.2% (4.4-6.1%)**	**14.7% (13.4-16.3%)**	**12.3% (11.3-13.7%)**
Caribbean	39.7	2.1 (1.81-2.36)	3.2 (2.8-3.7)	2.8 (2.5-3.1)	5.2% (4.5-5.9%)	8.1% (7.0-9.4%)	7.0% (6.3-7.7%)
Andean LA	52.7	2.3 (1.73-2.91)	10.6 (9.2-12.3)	10.3 (9.0-12.0)	4.3% (3.3-5.5%)	20.1% (17.5-23.3%)	19.6% (17.1-22.7%)
Central LA	230.3	13.5 (12.04-15.15)	41.8 (38.1-45.7)	44.0 (40.4-47.6)	5.9% (5.2-6.6%)	18.1% (16.6-19.9%)	19.1% (17.6-20.7%)
Southern LA	57.9	1.4 (1.00-1.92)	5.9 (5.1-7.0)	2.1 (1.5-2.7)	2.5% (1.7-3.3%)	10.2 (8.7-12.2%)	3.5% (2.5-4.8%)
Tropical LA	205.4	11.0 (6.83-15.77)	24.5 (18.0-32.5)	13.0 (8.1-19.4)	5.4% (3.2-7.7%)	11.9% (8.7-15.8%)	6.4 (3.9-9.5%)
**SSA**	**866.0**	**117.7 (111.1-125.9)**	**117.9 (108.7-127.1)**	**100.8 (94.1-108.0)**	**13.6% (12.9-14.6%)**	**13.6% (12.6-14.8%)**	**11.6% (10.9-12.6%)**
Central SSA	98.0	19.3 (16.5-22.2)	21.0 (17.8-24.7)	16.5 (13.6-20.1)	19.7% (16.6-22.6%)	21.4% (18.1-25.2%)	16.9% (13.9-20.5%)
East SSA	358.7	49.5 (45.7-54.3)	34.4 (30.3-38.8)	42.2 (37.9-46.8)	13.8% (12.8-15.2%)	9.6% (8.5-10.9%)	11.8% (10.6-13.1%)
Southern SSA	70.4	14.9 (12.9-17.3)	8.6 (6.7-10.7)	23.3 (20.7-26.0)	21.2% (19.1-25.8%)	12.2% (10.1-5.9%)	33.1% (30.8-38.7%)
West SSA	339.0	34.0 (30.0-38.9)	53.9 (46.7-60.7)	18.8 (15.3-23.2)	10.0% (8.9-11.5%)	15.9% (13.8-17.9%)	5.5% (4.5-6.8%)
**North Africa and Middle East**	**477.4**	**4.6 (4.0-7.1)**	**24.3 (22.6-28.5)**	**8.7 (7.3-10.7)**	**1.0% (0.9-1.6%)**	**5.4% (531–6.4%)**	**1.9% (1.6-2.4%)**
**Oceania**	**9.6**	**4.6 (4.3-3.8)**	**1.9 (1.6-2.2)**	**0.6 (0.6-0.7)**	**47.9% (44.7-51.0%)**	**19.7% (16.6-23.1%)**	**6.4% (5.8-7.0%)**
**GLOBAL**^**2**^	**5,631.4**	**438.9 (406.3-480.2)**	**819.0 (771.7-891.6)**	**464.6 (429.6-508.0)**	**7.8% (7.2-8.5%)**	**14.5% (13.7-15.8%)**	**8.3% (7.6-9.0%)**

^1^Credible interval, based on within-admin2 variation generated by Bayesian linear mixed model. LAC, Latin America and the Caribbean. SSA, sub-Saharan Africa.

^2^Global prevalence includes populations in Asia, LAC, SSA, North Africa and the Middle East and Oceania as the denominator.

**Figure 3:**
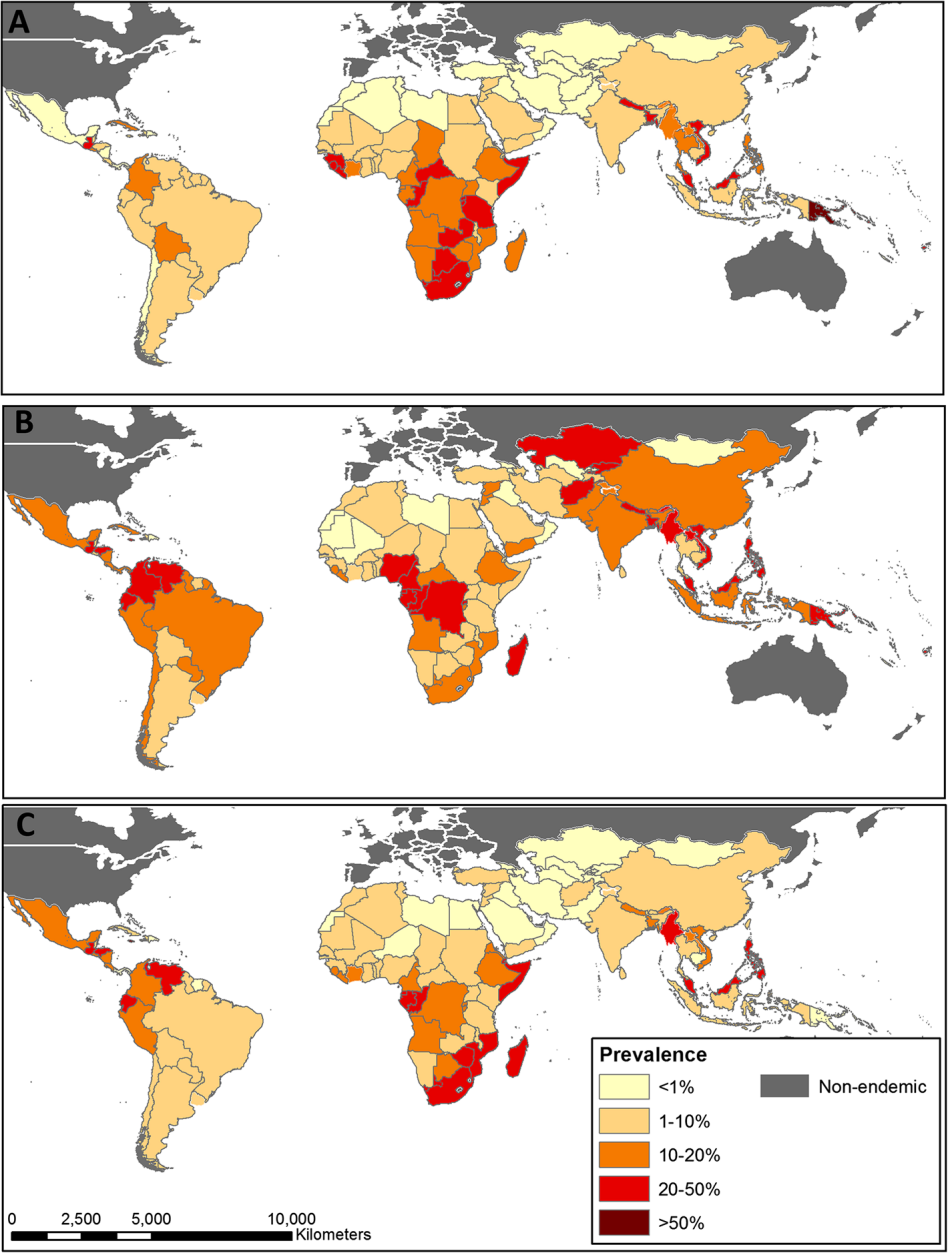
**Distribution of STH infection prevalence in 2010 by STH species. (A)** hookworm, **(B)***Ascaris lumbricoides* and **(C)***Trichuris trichiura*; based on geostatistical models for sub-Saharan Africa and available empirical information for all other regions.

The global distribution of each infection is shown in more detail in Figure 3. *A. lumbricoides* shows the widest distribution of the three STH, with the highest rates of transmission seen in Cameroon (national mean infection prevalence 30.8%), Nigeria (25.4%) and the north-western countries of Central sub-Saharan Africa (ranging from 32.2% in Congo to 38.8% in Equatorial Guinea), geographically dispersed countries in Asia (including Bangladesh 38.4%, Malaysia 41.7%, Afghanistan 36.0% and the Philippines 33.6%) and the southern countries of Central Latin America (Venezuela 28.4%, Colombia 26.0% and Ecuador 35.8%). *A. lumbricoides* is also common in the Central Asian countries of Kazakhstan (22.7%) and Kyrgyzstan (23.7%), and the Middle Eastern countries of Jordan, the Syrian Arab Republic, Yemen, the State of Palestine and Morocco (ranging from 8.0% in Morocco to 19.2% in Jordan). Hookworm infections remain common throughout much of sub-Saharan Africa (ranging from 2.3% in Eritrea to 30.5% in Central African Republic), in addition to Papua New Guinea (60.6%), Malaysia (21.0%), Nepal (30.7%) and Bangladesh (22.3%). In contrast, hookworm was not found in most of Central Asia and North Africa (excluding Egypt, where prevalence was 6.0%). Similarly, prevalence of *T. trichiura* was low in these regions. *T. trichiura* infections reach their highest prevalence in Malaysia (49.9%) and the Philippines (45.5%) as well as much of Central Africa (ranging from 11.8% in Central African Republic to 38.8% in Equatorial Guinea) and Central America (5.1% in El Salvador to 28.4% in Venezuela). Prevalence of hookworm was surprisingly low for India, at 7.9%, as observed previously [7]. In contrast, prevalence estimates for both hookworm and *A. lumbricoides* were high for Oceania, primarily driven by levels in Papua New Guinea.

**Figure 4:**
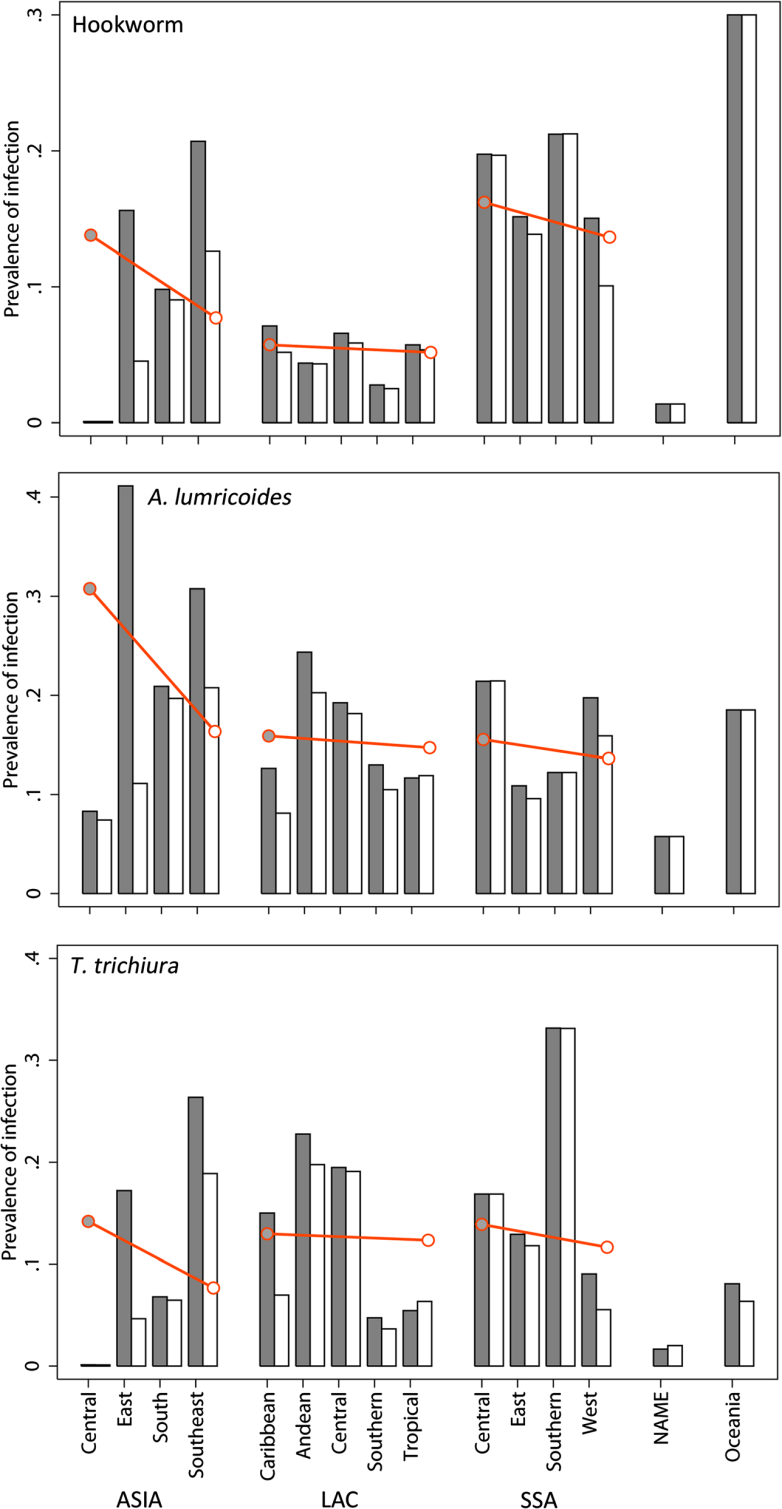
**Comparison of regional mean prevalence estimates for 2010 and 1990, by species.** Grey bars show sub-regional means for 1990, white bars sub-regional means for 2010; red line shows the change in overall regional mean prevalence between 1990 (filled circle) and 2010 (open circle).

Figure 4 shows regional changes in prevalence over the twenty year period for each species, by region and sub-region. The largest reductions over this time period are in Asia, where regional mean prevalence of hookworm dropped from 13.8 to 7.7%, *A. lumbricoides* from 30.8 to 16.3%, and *T. trichiura* from 14.2 to 7.7%. Much of this can be attributed to precipitous declines in the People’s Republic of China, where overall prevalence of any STH dropped from 57.5% in 1990 to 18.6% in 2010, and countries within southeast Asia (specifically Indonesia, which fell from 47.2 to 24.6%, Sri Lanka, which fell from 39.1 to 15.7%, and Thailand which fell from 38.4 to 21.3%). Reductions for other world regions are more modest: in Latin America prevalence of any STH infection fell by less than 3% from 29.2% to 27.4% and in sub-Saharan Africa by less than 5% from 36.8% to 32.2%).

### Global burden of STH infection

**Figure 5:**
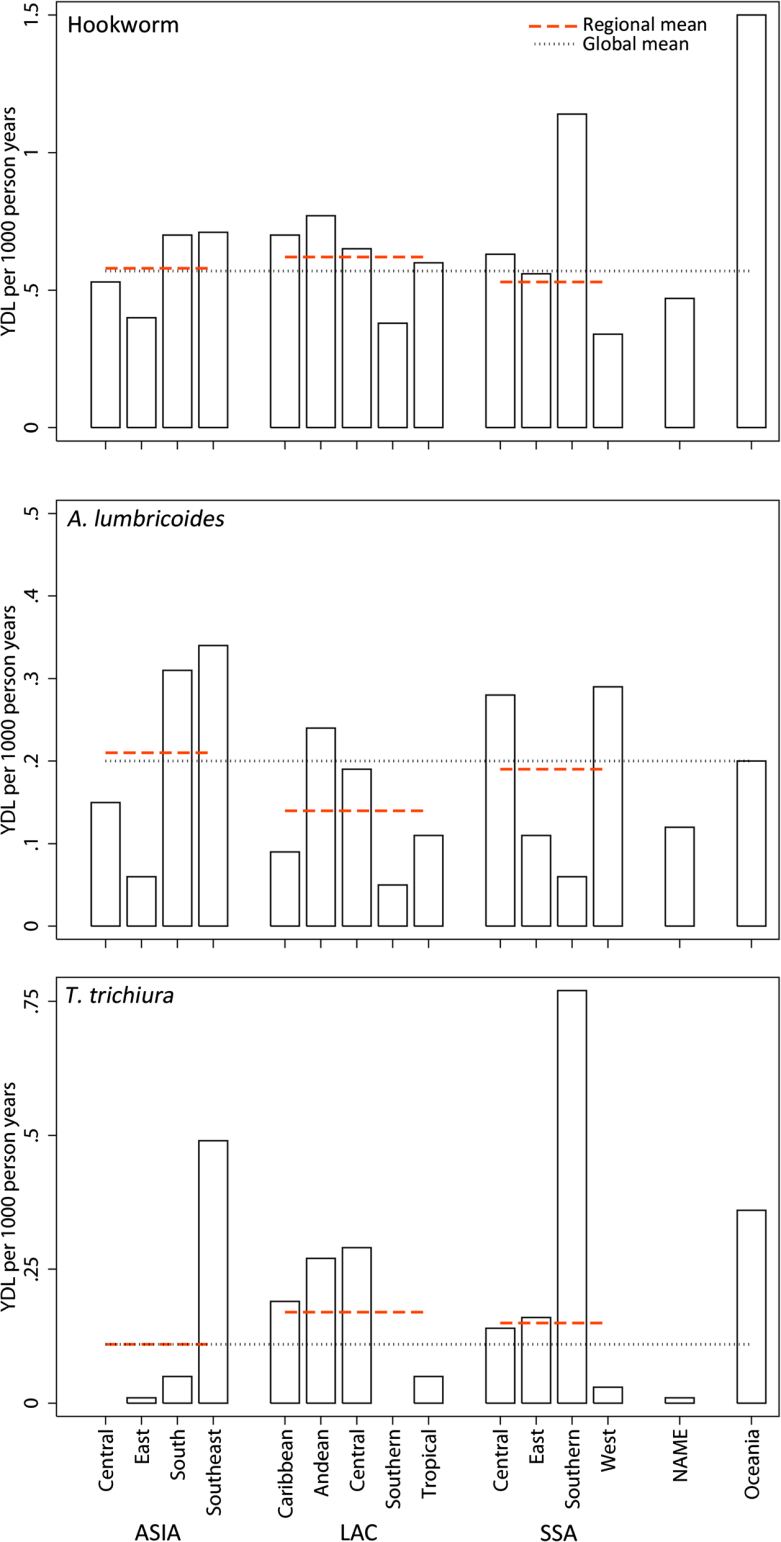
**Years Lived with Disability (YLD) per 1000 people for each region in 2010, by species.** Dashed red lines show regional means for Asia, Latin America and sub-Saharan Africa; dotted black line the global mean.

Regional YLD for each STH infection are taken from Murray *et al*. (2012) [50] and presented in Table 5. Of the 4.98 million YLDs attributable to STH globally in 2010, 65% are attributable to hookworm, 22% to *A. lumbricoides* and the remaining 13% to *T. trichiura.* The vast majority of these occur in Asia: 35% of YLDs attributable to hookworm and 45% of those attributable to *A. lumbricoides* are lost by populations in south Asia and 47% of those attributable to *T. trichiura* by southeast Asian populations. In relative terms however, the distribution of disability attributable to STH varies more considerably within major global regions than between them, especially for *A. lumbricoides*, as is highlighted in Figure 5. Highest rates for hookworm are observed in southern sub-Saharan Africa (1.14 YLD / 1000 people) and Oceania (2.10 YLD / 1000 people), whilst for *A. lumbricoides* the highest rates are seen in south and southeast Asia (0.31-0.34 YLD / 1000 people) and west sub-Saharan Africa (0.29 YLD / 1000 people). southeast Asia (0.49 YLD / 1000 people) and southern sub-Saharan Africa (0.77 YLD / 1000 people) experience the highest relative burden for *T. trichiura*. These remarkable differences in the relative burden of each infection within regions, most noticeable for *T. trichiura* in southern sub-Saharan Africa and southeast Asia, are a consequence of moderate differences in estimated prevalence and the non-linear relationship between prevalence and intensity (and thus morbidity).

**Table 5:** Estimates years lived with disability (YLDs) due to STH in 2010, by region

**Region**	**Hookworm**		***A. lumbricoides***		***T. trichiura***	
	** YLDs**	**% total**	** YLDs**	**% total**	** YLDs**	**% total**
**Asia**	**2,176,895**	**67.4%**	**801,830**	**72.2%**	**397,353**	**62.3%**
Central	43,086	1.3%	11,986	1.1%	-	0.0%
East	568,112	17.6%	79,932	7.2%	18,199	2.9%
South	1,130,070	35.0%	499,599	45.0%	81,681	12.8%
Southeast	435,627	13.5%	210,314	18.9%	297,473	46.6%
**Latin America (LA) and the Caribbean**	**364,962**	**11.3%**	**83,776**	**7.5%**	**100,126**	**15.7%**
Caribbean	27,655	0.9%	3,553	0.3%	7,570	1.2%
Andean	40,790	1.3%	12,563	1.1%	14,141	2.2%
Central	150,274	4.7%	43,178	3.9%	67,207	10.5%
Southern	22,043	0.7%	2,616	0.2%	89	0.0%
Tropical	124,199	3.8%	21,865	2.0%	11,120	1.7%
**Sub-Saharan Africa (SSA)**	**456,823**	**14.1%**	**168,652**	**15.2%**	**134,055**	**21.0%**
Central	61,461	1.9%	27,512	2.5%	14,143	2.2%
East	200,405	6.2%	38,266	3.4%	56,994	8.9%
Southern	80,035	2.5%	4,006	0.4%	54,430	8.5%
West	114,922	3.6%	98,868	8.9%	8,487	1.3%
**North Africa and the Middle East**	**211,940**	**6.6%**	**54,466**	**4.9%**	**3,223**	**0.5%**
**Oceania**	**20,180**	**0.6%**	**1,876**	**0.2%**	**3,443**	**0.5%**
***GLOBAL***	***3,230,800***		***1,110,600***		***638,200***	

There were an estimated 2,824 deaths attributable to *A. lumbricoides* in 2010, with most occurring in populations from Asia and south Asia, increasing the global DALYs attributable to *A. lumbricoides* to 1.31 million (0.71 – 2.35 million). For hookworm and *T. trichiura* – for which no deaths are attributed – the YLDs represent the total DALYs contributed by these infections. This brings the DALYs contributed by STH to 5.18 million in 2010, with 3.23 caused by hookworm, 1.31 by *A. lumbricoides* and 0.64 by *T. trichiura*. Figures for 1990 are much higher (9.01 million in total): hookworm was estimated to contribute 3.93 million DALYs, *A. lumbricoides* 4.22 million and *T. trichiura* 0.86 million.

## Discussion

It is widely acknowledged that the exact global numbers infected and experiencing morbidity attributable to STH infection will remain an elusive goal, due in part to a paucity of reliable and accurate epidemiological data and in part the non-specificity of clinical signs due to STH [7,8,30,58]. Here, we build upon a modelling framework originally proposed by Chan *et al*. for use in the first GBD study [5] to provide an update on the global situation in 2010. We estimate that 1.45 billion people were infected worldwide with at least one species of intestinal nematode in 2010, resulting in 4.98 million YLD and 5.18 million DALYs. The vast majority of infections and burden occurred in Asia, where at least one quarter (26.4%) of the population were thought to host at least one STH species. In relative terms however, morbidity attributable to STH infection was more equal across regions, typically falling between 0.6 and 1.4 YLD per 1000 people. Placing these results in context, the GBD 2010 study estimated that the neglected tropical diseases (NTDs) were together attributable for 26.05 million DALYs (1% of all DALYs), and communicable diseases more generally 1.34 billion DALYs (54% of all DALYs) [50].

Analysis of trends between 1990 and 2010 highlight a number of interesting findings. Overall, prevalence of any STH across all endemic regions has dropped from 38.6% in 1990 to 25.7% in 2010, representing a reduction of 140 million infected individuals. Steep declines were seen in countries such as the People’s Republic of China, Indonesia and Republic of Korea, but declines were more modest in other Asian countries and in sub-Saharan Africa and Latin America and the Caribbean. Reductions in DALYs were notably bigger, owing to the non-linear relationship between overall infection prevalence and prevalence of high intensity infection [15]. This highlights the substantial public health gains that have been made over the past 20 years, with sizeable reductions in the number of children suffering the wasting, anaemia and abdominal pain associated with high intensity STH infection.

Our current estimates differ from those produced previously: the first GBD study estimated that in 1990 hookworm prevalence across all endemic regions was 30%, *A. lumbricoides* was 33.5% and *T. trichiura* was 24.4%, resulting in an estimated 2.52 billion infections worldwide [58], nearly double the 1.45 billion predicted here for the same year. Prevalence estimates by de Silva *et al*. in their 2003 update are also substantially higher at 2.15 billion [7]. These discrepancies can be credited to a number of methodological improvements. First, by applying environmental limits we were able to shrink national populations at-risk to include only those living in areas where transmission of infection was plausible [11], thus preventing prevalence assignment to populations living in environmentally inhospitable regions within endemic countries (122–125 million people globally, depending upon species). Second, our estimates have been defined using data from the updated GAHI [20], and thus represent a much larger database than those used in previous estimates. For example, de Silva *et al*. identified 494 publications with suitable data, in comparison to the 862 publications included in the current analysis. They also relied on much older data (dating back to 1960s or before) when estimating infection prevalence for many Latin American countries, which may partly explain the large differences in 1990 and 1994 estimates from the two analyses. Third, geographical variation of both worms and population within countries were handled more robustly and at higher spatial resolution than previous estimates. For countries outside of sub-Saharan Africa, empirical estimates were applied to the admin2 level (where available) and aggregated to generate population-weighted national estimates, thus potentially preventing unrepresentative point prevalence estimates unduly influencing national estimates; though some countries still lacked suitable data. Within sub-Saharan Africa, Bayesian geostatistical modeling was used to predict the prevalence of infection for 2010, using available data and environmental information. This allowed more accurate predictions to be made for areas with no available survey data.

Reliable estimates of prevalence depend crucially on sampling methods and diagnosis, and thus our estimates inevitably come with some important caveats. First, we emphasize that these results do not all derive from nationally representative, spatially random surveys. Whilst for the majority of countries the total sample size used was at least several thousand individuals, for many epidemiologically important regions (including much of sub-Saharan Africa and south and southeast Asia) data were insufficient. This in part explains higher than expected estimates for Oceania, which in the absence of additional data were driven by evidence of very high prevalence of hookworm infection in Papua New Guinea [59,60]. The seemingly anomalous high STH prevalence seen in Malaysia (and consequently the large relative burden in terms of YLD/person shown for southeast Asia in Figure 5) can also be ascribed to the few available data, this time from high risk communities in Sarawak [61], Pulau Pinang [62], Selangor [63,64] and Kelantan [65] and is unlikely to be truly representative of the whole population. This scarcity of data for many Asian countries (and also reliance upon national surveys) can be, at least in part, attributed to necessary restriction of the literature search to English, French and Spanish language sources. However, the national surveys used for the People’s Republic of China, and the Republic of Korea, were powered at the province level and therefore were appropriate for our needs. We thus preferred to give emphasis to the national survey compared to small-scale surveys included in the local literature. By way of comparison, a recent systematic review and associated geostatistical model for China developed by Vounatsou *et al.* predicted a lower national combined STH prevalence (11% compared with 18% seen here), a consequence of lower predicted STH prevalence in Northern and Eastern Provinces [66]. Lastly, for eight countries (Armenia, Bahrain, Kuwait, Lebanon, Qatar, Syrian Arab republic, United Arab Emirates and western Sahara) no data were available, and it was necessary to rely on data from neighbouring countries with similar environmental and socio-economic conditions. Whilst this risks misestimation of national prevalence for these countries, all these countries are considered very low risk and it is likely that the total populations infected, and hence potential error incurred, will be small.

For sub-Saharan Africa, we were able to overcome sparse availability by modelling prevalence using an MBG framework. This combined empirical and geostatistical modelling approach is now becoming more widely applied in parasitology and infectious disease epidemiology more generally [67], and has been used for example to map the distribution of not only STH, but also schistosomiasis, leishmaniasis, malaria, trachoma, lymphatic filariasis and loaiasis at national, regional or continental scales [24,68-76]. The generated predictive maps for sub-Saharan Africa only contained information on survey prevalence and environmental and population covariates however, and did not account for other important proximal risk factors including access to clean water and adequate sanitation facilities [77,78] and implementation of control [79-82]. Results for southern Africa, for example, have been driven by the few available data-points in high prevalence regions, namely low-lying surrounding areas of Cape Town in western Cape Province and the sub-tropical lowlands of KwaZulu-Natal Province, which were collected in informal settlements with poor sanitation and high population crowding [83-92]. This explains in part the very high relative *T. trichiura* and hookworm burden (in YLD/person) demonstrated for these southern sub-Saharan Africa countries. We were also unable to incorporate measures of uncertainty originating from this model as a single point prediction process was used and thus aggregated certainty estimates would have been invalid. This unfortunately means that the high levels of prediction uncertainty that exist at prediction locations far from any available survey data (that is, for those countries with little or no data) are not reflected by the credible intervals presented in Table 4 We are now working to improve the continental STH map for sub-Saharan Africa by including information on these important covariates and better accounting for geostatistical outliers.

Data may also be systematically biased if survey sites were purposely chosen in areas of known high infection risk. Reassuringly however, 644 (9.7%) of survey sites were negative for all three STH species, and 3185 (47.7%) were negative for at least one species, suggesting that this concern may in general be unfounded. Urban–rural disparities in infection prevalence are also likely to have an impact on national mean prevalence globally, although observed relationships are too inconsistent for us to confidently apply corrections to national prevalence data at global scales [11]. Finally, data were available for only limited time points for many countries. In the absence of a consistent, measurable temporal trend we had to assume that prevalence had not changed between 1990–1999 and 2000–2010 for 70 countries with insufficient data, with prevalence estimates adjusted only for those 30 countries that have implemented large-scale treatment campaigns 2005–2010. However, evidence from Kenya does suggest that STH prevalence may have reduced in the past two decades, even in the absence of large-scale control or major improvements in sanitation [24].

In addition to prevalence, we also present YLD and DALYs for STH produced by the core GBD 2010 team [50,51]. These estimates are intended to supersede those published previously, and are not directly comparable. Details of the methods used are provided elsewhere [50-52]; however, some issues should be highlighted when reflecting upon the derived estimates. There are a number of major methodological improvements: disability weights were derived from judgments of the general public about the health loss associated with the health state related to each sequela, rather than by health care professionals alone [52]; discounting and age-weighting were removed when estimating DALYs. For hookworm, GBD estimates previously only linked anaemia to high intensity infection. However, based on a recent summary of available evidence which showed that even light infections were associated with lower haemoglobin concentration in adults [44], in this current analysis anaemia outcomes attributable to hookworm infection were applied to all infected individuals regardless of infection intensity.

Controversially however, cognitive impairment has been removed as a sequela for STH, justified by the perceived paucity of evidence of a cognitive impact of intestinal nematodes. This judgment was made primarily on the basis of a 2012 Cochrane review of available randomized controlled trials of deworming, which found contrasting evidence of nutritional benefits and little support for cognitive or education benefits [93]. Critics of this review have argued that not only did this review exclude studies that treated both STH and schistosomiasis, but also that many of the underlying trials of deworming suffer from a number of methodological challenges, non-assessment of treatment externalities, inadequate measurement of cognitive outcomes and school attendance, and sample attrition, and that further, better-designed studies are required [94]. Such studies should ideally be randomized, must be appropriately powered and have sufficient duration of follow-up, care should be taken to collect detailed information on all potential confounders, and outcome measures should include all mediating variables along the hypothesized causal chain from deworming, anaemia, sustained attention and educational development. Further, whilst analysis did take into account the underlying distributions of wasting and haemoglobin density within populations, sparse and low spatial resolution data for many world regions will have lead to inaccurate YLD calculation for STH and other associated conditions.

There are additional limitations specific to the STH burden estimates. Firstly, the indirect methods used to estimate the proportion of the infected population experiencing high intensity infections (and thus at risk of morbidity) are highly sensitive to the choice of model parameters used to define the fitted negative binomial distribution and the chosen intensity cut-offs. In the absence of additional empirical data to better define these relationships, we were limited to using the same parameters and thresholds for *A. lumbricoides* and *T. trichiura* as previous efforts [5,30]. Although additional data for hookworm did allow us to better quantify associations here, these primarily originate from school-aged children living in sub-Saharan Africa and so observed relationships may not be generalisable to other population groups and settings. Future work will concentrate on better quantifying these relationships, and incorporating uncertainty in resulting models. Similarly, mortality estimates for *A. lumbricoides* are subject to considerable ambiguity since the data sources by which the GBD estimates are derived are unclear.

More generally, the use of DALYs as a measure of disease burden has its advantages and its disadvantages [95]. The main advantage is that DALYs provide a composite, internally consistent measure of population health which can be used to evaluate the relative burden of different diseases and injuries and compare population health by geographic region and over time. Combined with information on the effectiveness and cost of different interventions, such estimates can guide priority setting [96]. The main disadvantage of DALYs is that they focus solely on health and do not capture the broader societal impact of diseases. This is especially true for STH, which have subtle, lasting impacts on child development and education. For example, recent studies in Africa, as well as reanalysis of the extensive Rockefeller Foundation supported efforts to control hookworm in the southern United States at the beginning of the 20th century, have shown remarkable long-run effects on productivity and employment and wages of treating children at school age [97-99]. There is also an important equity issue that goes beyond health: STH and other NTDs affect the poorest communities and are diseases of neglected populations. Thus, tackling STH and other NTDs should be seen part of broader efforts to reduce global poverty.

## Conclusion

Improvements in the cartography of helminth infection have enabled us to better estimate the public health burden of STH. The updates presented here, based upon informed approximation using available data, clearly indicate that STH infections remain highly endemic throughout much of the tropics and sub-tropics. Although there have been substantial declines in some regions, in general infection levels over the past twenty years have remained unacceptably high. Whilst it is clear that we still require a better understanding of the full burden of intestinal nematodes, including effects upon child development and cognition, the extraordinary number of STH infections and associated years lost due to disability has helped galvanise the NTD community into reducing the burden of STH infections. Given the current high profile of NTDs on the global public health agenda, and the availability of simple, low-cost interventions that can substantially control the morbidity due to these infections, it’s our collective responsibility to ensure that the next set of STH distribution and burden estimates prove more encouraging.

### Endnotes

^a^Countries with no data, transmission excluded for socio-economic reasons: Armenia, Anguilla, Antigua and Barbuda, Aruba, Bahamas, Bahrain, Barbados, Bermuda, British Virgin Islands, Cayman Islands, Falkland Islands, Guadaloupe, Kuwait, Lebanon, Montserrat, Netherlands Antilles, Qatar, Syrian Arab Republic, Turks and Caicos Islands, United Arab Emirates, western Sahara.

^b^Oceania countries with no data assigned regional mean prevalence: Guam, New Caledonia, Northern Mariana Islands, Palau, Pitcairn Islands, Tokelau, Wallis and Futuna.

## Competing interests

The authors declare that they have no competing interests.

## Authors’ contributions

Conceived the study: RLP and SJB contributed to data assembly: JLS and SJB Designed and performed the analyses: RLP, RJ. Wrote the first draft of the manuscript: RLP Contributed to the writing of the manuscript: SJB. All authors read and approved the originally submitted and the revised manuscript.

## Supplementary Material

Additional file 1 Geostatistical estimation of STH prevalence across SSA. Click here for file

Additional file 2 Breakdown summary of included data sources, by country. Click here for file
