# Supplementary material for: Global numbers of infection and disease burden of soil transmitted helminth infections in 2010
Source: Parasit Vectors. 2014 Jan 21;7:37. doi: 10.1186/1756-3305-7-37 (PMC3905661; doi:10.1186/1756-3305-7-37)
Supplement: Additional file 1 — Geostatistical estimation of STH prevalence across SSA. [file 1756-3305-7-37-S1.docx]

# Additional File 1 - Geostatistical estimation of STH prevalence across SSA

This document outlines the analysis strategy used to develop the risk maps of soil-transmitted helminth infection (STH) for sub-Saharan Africa (SSA) subsequently used in the estimation of the global numbers infected and disease burden.

#### Analysis outline

The objective of these analyses was to determine the spatial distribution of STH infection prevalence across SSA. Prevalence data for STH (hookworm, *Ascaris lumbricoides* and *Trichuris trichiura*) were collated using search principles and criteria outlined below, in order to create a robustly geo-located dataset of helminth surveys. This database was used to make a continuous cumulative prevalence surface adopting a Bayesian space-time geostatistics approach, adjusting for environmental covariates; no spatial prediction was made for areas masked as environmentally unsuitable for STH transmission. The resulting models were used to interpolate the prevalence of infection in school-aged children (5-14 years) across SSA. The age-stratified population infected with each species was then extracted by second administrative level, as described in the manuscript.

#### Data sources

Survey data were identified through structured searches of electronic bibliographic databases, complemented with manual searches of local archives and libraries and direct contact with researchers. References from identified publications were checked for additional surveys. Estimates of infection prevalence were included according to pre-defined criteria: only cross-sectional prevalence surveys were included; data were excluded if based on hospital or clinic surveys, post-intervention surveys, or surveys among sub-populations, such as among refugees, prisoners or nomads. In instances where multiple surveys from the same location were surveyed at different times, each survey was included. Abstracted data included details on the source of the data, date and location of survey, characteristics of the surveyed population, survey methodology, method of diagnosis, age range of sampled individuals, and the number of individuals examined and the number positive with hookworm, A. lumbricoides and T. trichiura. Authors of published data were contacted if relevant information was unclear from the original reports. For the current analysis, survey data were collected between 1974 and present day.

The longitude and latitude of each survey were determined using a combination of resources including a national schools databases, village databases digitised from topographical maps and a range of electronic gazetteers (see Brooker et al. [[1](#_ENREF_1)]); and contact with authors who used GPS.

#### Ecological and climatic covariates and limits of transmission

Normalised differenced vegetation index (NDVI; a measure of vegetation density) and land surface temperature (LST) based on the period 1992 to 1996 at 5km resolution were obtained for the National Oceanographic and Atmospheric Administration’s (NOAA) Advanced Very

High Resolution Radiometer (AVHRR) (<http://noaasis.noaa.gov/NOAASIS/ml/avhrr.html>). Temporal Fourier analysis (TFA) was used, and Fourier Processed products (annual amplitude) were assembled [[2](#_ENREF_2)]. Population density was derived from adjusted population counts for the year 2000 projected to 2009 by applying national, medium variant, inter-censal growth rates [[3](#_ENREF_3)]. The annual amplitude products for LST and NDVI were standardised to optimise sampling during MCMC by subtracting the arithmetic mean and dividing by the standard deviation.

These ecological data, along with results from previous studies, were used to define the spatial limits for the transmission of STH. Specifically, it has been shown experimentally that the development of free-living infectious stages of *A. lumbricoides* and *T. trichiura* ceases at 38^o^C and hookworm at 40^o^C [[4-7](#_ENREF_4)]. This is supported by an observed relationship between prevalence across sub-Saharan Africa and annual amplitude products for LST and NDVI. On this basis, areas were masked as unsuitable for STH transmission where the annual amplitude products for LST and NDVI exceeded extreme limits (i.e. too hot and/or arid). No spatial prediction was subsequently made for such areas.

#### Bayesian space-time modelling approach

The probability models used in this study assumed that individuals participating in each sample were egg-positive for helminth infection with a probability that was a continuous function of the time and location of the survey, modified by a set of covariates, and modelled as a Gaussian process [[8](#_ENREF_8)]. The Bayesian space-time model was implemented in two parts starting with an inference stage in which a Markov Chain Monte Carlo (MCMC) algorithm was used to generate samples from the join posterior distribution of the parameter set and the space-time random field at the data locations. This was followed by a prediction stage in which samples were generated from the posterior distribution of infection prevalence at each prediction location on a 5 x 5 km grid. Each species was modelled separately. Both the inference and prediction stages were coded using Python (PyMC version 2.0) [[9](#_ENREF_9)]

For each species, the *N_i_* individuals included in survey *i* were assumed egg-positive with probability $P(x_{i},t_{i})$, so that the number positive (*Y_i_*) was distributed binomially:

$$Y_{i}|N_{i},P(x_{i},t_{i})\sim\mathrm{Binomial}(N_{i},P(x_{i},t_{i}))$$

The coefficient $P(x_{i},t_{i})$ at location *x* and time *t* was modelled as the inverse logit function applied to a space-time component, plus an unstructured (random) component. The unstructured component $\varepsilon(x_{i})$ was represented as a Gaussian process with zero mean and variance *V*. The space-time component was represented by a stationary Gaussian process $f\left( x,t \right)$ with mean $\mu$ and covariance *C.*

The mean component was modelled as a linear function of standardised maximum LST and NDVI, whether the prediction location $x$ was extreme rural (defined as <10 persons per km^2^) and whether the survey was community-based rather than school-based. The mean component was therefore defined by x parameters:

$$\mu= \beta_{x}+\sum_{k=1}^{K} \beta_{k}X_{i.x,t,k}$$

where $\sum_{k=1}^{K} \beta_{k}X_{i.x,t,k}$ denotes the matrix of included covariates and $\beta_{x}$ the intercept.

Covariance between spatial and temporal locations was modelled using the space-time covariance function *C*:

$$C\left( x_{i},t_{i},x_{j,}t_{j} \right)= \tau^{2}\gamma\left( 0 \right)\frac{\left( \Delta x \right)^{\gamma\left( \Delta t \right)}K_{\gamma\left( \Delta t \right)}\left( \Delta x \right)}{2^{\gamma\left( \Delta t \right)-1}\Gamma\left( \gamma\left( \Delta t \right)+1 \right)}$$

$$\gamma\left( \Delta t \right)= \frac{1}{2\rho+2\left( 1-\rho\right)\left[ \left( 1-\upsilon\right)e^{-\left| \Delta t \right|/\phi t}+\upsilon\cos(2\pi\Delta t) \right]}$$

$$\Delta t=|t_{i}- t_{j}$$

$K_{\gamma}$ is the modified Bessel function of the second kind of order $\gamma$, and $\Gamma$ is the gamma function.

Spatial distances between a pair of points $x_{i}$ and $x_{j}$ was computed as the great-circle distance $D_{GC}(x_{i},x_{j})$ multiplied by a factor that depends on the angle of inclination $\theta(x_{i},x_{j})$ of the vector pointing from $x_{i}$ to $x_{j}$. $\theta$ was computed as if latitude and longitude were Euclidean coordinates (on a cylindrical projection):

$$\Delta x=2\sqrt{\gamma(\Delta t)}\frac{D_{GC}(x_{i},x_{j})\sqrt{1-\psi^{2}\cos^{2}(\theta(x_{i},x_{j})- \lambda)}}{\phi_{x}}$$

As temporal separation increases, the covariance approaches a limiting sinusoid

$\tau^{2}\left[ \rho+\left( 1-\rho\right)\upsilon\cos\left( 2\pi\Delta t \right) \right]$ rather than sero. On the other hand, when $\Delta t=0$ (i.e. points at different locations but the same time), this reduced to a standard exponential form with a range parameter $\phi_{x}\sqrt{2}$.

The square root of the partial sill $\tau$ and the spatial range parameter $\phi_{x}$were assigned skew-normal priors. An exponential, proper prior was assigned to $\phi_{t}$, and a uniform prior was assigned to the direction of anisotropy parameter $\lambda$ and to the the “eccentricity” parameter $\psi^{2}$, which control the amount of anisotropy. A uniform prior was assigned to the limiting autocorrelation in the temporal direction, $\rho$, and a standard prior was assigned to the components of the mean.

#### Model implementation and output

Bayesian inference was implemented using Markov Chain Monte Carlo to generate samples from the posterior distribution of the Gaussian field at each data location and of the unobserved parameters of the mean, covariance function and Gaussian random noise component.

For each species, samples were generated from the mid-year 2010 mean of the posterior distribution of at each prediction location at points on a regular 5 × 5 km spatial grid across sub-Saharan Africa. Model output therefore consisted of samples from the predicted posterior distribution of the 2010 infection prevalence at each grid location, which were used to generate point estimates of infection prevalence.

#### References

1. Brooker S, Kabatereine NB, Smith JL, Mupfasoni D, Mwanje MT, Ndayishimiye O, Lwambo NJS, Mbotha D, Karanja P, Mwandawiro C *et al*: **An updated atlas of human helminth infections: the example of East Africa**. *Int J Health Geogr* 2009, **8**:42.

2. Hay SI, Tatem AJ, Graham AJ, Goetz SJ, Rogers DJ: **Global Environmental Data for Mapping Infectious Disease Distribution**. *Adv Parasitol* 2006, **62**:37-77.

3. Hay SI, Guerra CA, Gething PW, Patil AP, Tatem AJ, Noor AM, Kabaria CW, Manh BH, Elyazar IR, Brooker S *et al*: **A world malaria map: *Plasmodium falciparum* endemicity in 2007**. *PLoS Med* 2009, **24**(6):e1000048.

4. Beer RJ: **The relationship between *Trichuris trichiura* (Linnaeus 1758) of man and *Trichuris suis* (Schrank 1788) of the pig**. *Res Vet Sci* 1976, **20**(5):47-54.

5. Seamster AP: **Developmental studies concerning the eggs of *Ascaris lumbricoides* var. *suum*.** *Am Mid Natur* 1950, **43**:450-468.

6. Udonsi JK, Atata G: ***Necator americanus*: Temperature, pH, Light, and Larval Development, Longevity, and Desiccation Tolerance**. *Exp Parasitol* 1987, **63**:136-142.

7. Smith G, Schad GA: ***Ancylostoma duodenale* and *Necator americanus*: effect of temperature on egg development and mortality**. *Parasitol* 1989, **99**(1):127-132.

8. Banderjee S, Carlin BP, Gefland AE: **Hierarchical modeling and analysis for spatial data**. Boca Ranton, Florida, USA: Chapman and Hall / CRC Press LLC; 2004.

9. Patil AP, Huard D, Fonnesbeck CJ: **PyMC: Bayesian stochastic modelling in Python**. *J Stat Soft* 2010, **35**(4).
